# A genetic network of flowering-time genes in wheat leaves, in which an *APETALA1*/*FRUITFULL*-like gene, *VRN1*, is upstream of *FLOWERING LOCUS T*

**DOI:** 10.1111/j.1365-313X.2009.03806.x

**Published:** 2009-02-26

**Authors:** Sanae Shimada, Taiichi Ogawa, Satoshi Kitagawa, Takayuki Suzuki, Chihiro Ikari, Naoki Shitsukawa, Tomoko Abe, Hiroyuki Kawahigashi, Rie Kikuchi, Hirokazu Handa, Koji Murai

**Affiliations:** 1 Department of Bioscience, Fukui Prefectural UniversityEiheiji-cho, Fukui 910-1195, Japan; 2 Plant Genome Research Unit, National Institute of Agrobiological SciencesTsukuba, 305-8602, Japan; 3 RIKEN Nishina CenterWako, Saitama 351-0198, Japan; 4 Graduate School of Life and Environmental Sciences, The University of TsukubaTsukuba, 305-8572, Japan

**Keywords:** flowering, vernalization, photoperiod, wheat, genetic network

## Abstract

To elucidate the genetic mechanism of flowering in wheat, we performed expression, mutant and transgenic studies of flowering-time genes. A diurnal expression analysis revealed that a flowering activator *VRN1*, an *APETALA1*/*FRUITFULL* homolog in wheat, was expressed in a rhythmic manner in leaves under both long-day (LD) and short-day (SD) conditions. Under LD conditions, the upregulation of *VRN1* during the light period was followed by the accumulation of *FLOWERING LOCUS T* (*FT*) transcripts. Furthermore, *FT* was not expressed in a *maintained vegetative phase* (*mvp*) mutant of einkorn wheat (*Triticum monococcum*), which has null alleles of *VRN1*, and never transits from the vegetative to the reproductive phase. These results suggest that *VRN1* is upstream of *FT* and upregulates the *FT* expression under LD conditions. The overexpression of *FT* in a transgenic bread wheat (*Triticum aestivum*) caused extremely early heading with the upregulation of *VRN1* and the downregulation of *VRN2*, a putative repressor gene of *VRN1*. These results suggest that in the transgenic plant, *FT* suppresses *VRN2* expression, leading to an increase in *VRN1* expression. Based on these results, we present a model for a genetic network of flowering-time genes in wheat leaves, in which *VRN1* is upstream of *FT* with a positive feedback loop through *VRN2*. The *mvp* mutant has a null allele of *VRN2*, as well as of *VRN1*, because it was obtained from a spring einkorn wheat strain lacking *VRN2*. The fact that *FT* is not expressed in the *mvp* mutant supports the present model.

## Introduction

Floral transition, the phase transition from vegetative to reproductive development, is a critical event in the life cycle of seed-propagated plants. In Arabidopsis, the current consensus is that there are four major flowering promotion pathways: the vernalization, photoperiod, autonomous and gibberellin (GA) pathways (Boss *et al.*, 2004). The vernalization and photoperiod pathways integrate environmental signals into the floral transition, whereas the autonomous and GA pathways act independently of external signals. The vernalization pathway mediates the promotion of floral transition, and is induced by low temperatures. *FLOWERING LOCUS C* (*FLC*), a MADS-box gene that acts as a repressor of the floral transition, is negatively regulated by vernalization (Michaels and Amasino, 1999; Sheldon *et al.*, 1999). Vernalization results in the stable reduction of the levels of the flowering repressor *FLC* by epigenetic regulation (Sung and Amasino, 2006). The autonomous pathway prevents the accumulation of *FLC* mRNA by epigenetic regulation (He and Amasino, 2005), indicating that the vernalization and autonomous pathways are connected through the flowering repressor *FLC* (Jiang *et al.*, 2008). The photoperiod pathway, which consists of photoreceptor, circadian clock and circadian clock-regulated genes, promotes the floral transition in response to a long photoperiod (Searle and Coupland, 2004). This pathway includes downstream genes of the circadian clock, such as *CONSTANS* (*CO*) and *FLOWERING LOCUS T* (*FT*).*CO* encodes a transcription factor with two B-box zinc fingers, and directly induces *FT* expression (Putterill *et al.*, 1995). Circadian clock-regulated *CO* transcription is mediated by controlling factors such as GIGANTEA (GI) proteins (Imaizumi and Kay, 2006). In long-day (LD) conditions *CO* expression peaks during the light period, resulting in the activation of *FT* expression, thereby causing early flowering (Yanovsky and Kay, 2003). *FT* encodes a protein, similar to the animal Raf kinase inhibitor-like protein, that functions as a flowering promoter (Kardailsky *et al.*, 1999; Kobayashi *et al.*, 1999). *FT* is an integrator of the vernalization and photoperiod pathways: prior to vernalization, the activity of *FT* is directly suppressed by *FLC* (Searle *et al.*, 2006). Recent studies indicate that the FT protein functions as a systemic signaling molecule from leaf to apex (Corbesier *et al.*, 2007; Jaeger and Wigge, 2007; Mathieu *et al.*, 2007). The FT protein interacts with the bZIP transcription factor FD at the apex, and activates the floral meristem identity genes *APETALA1* (*AP1*) and *LEAFY* (*LFY*) (Abe *et al.*, 2005; Wigge *et al.*, 2005). The growth regulator GA promotes flowering by upregulating the transcription of *SUPPRESSOR OF OVEREXPRESSION OF CO1* (*SOC1*), an activator of *LFY* (Moon *et al.*, 2003).

In cereal crops such as wheat and barley, the heading time is associated with the timing of floral transition, and is an important character because of its influence on adaptability to various environmental conditions. Bread wheat (*Triticum aestivum*, 2n=6x=42, genome constitution AABBDD) is grown in a wide range of environments, all over the world, and its wide adaptability results from the variation in heading time among cultivars. Many genetic studies have been performed to clarify the genetic control of heading time in wheat, and the following three component characteristics have been identified: vernalization requirement, photoperiod sensitivity and narrow-sense earliness (earliness *per se*) (Worland and Snape, 2001).

Vernalization requirement is concerned with the sensitivity of the plant to cold temperatures for accelerating spike primordium formation. The vernalization insensitivity (spring habit) genes, *Vrn-A1*, *Vrn-B1* and *Vrn-D1*, have been shown to be located on chromosomes 5A, 5B and 5D, respectively, of bread wheat (Worland and Snape, 2001). Bread wheat is a hexaploid species with the genome constitution AABBDD that originated from three diploid ancestral species: the A genome came from *Triticum urartu*, the B genome came from *Aegilops speltoides*, or another species classified in the Sitopsis section, and the D genome came from *Aegilops tauschii* (Feldman, 2001). Consequently, the hexaploid wheat genome contains triplicated homoeologous genes (homoeologs) derived from the ancestral diploid species. *Vrn-A1*, *Vrn-B1* and *Vrn-D1* are three homoeologs of the *Vrn-1* gene. Yan *et al.* (2003) isolated *Vrn-A*^*m*^*1*, an ortholog of *Vrn-A1*, in diploid einkorn wheat *Triticum monococcum* (2n=2x=14, A^m^A^m^) using a map-based method, and named this gene *VRN1. VRN1* was shown to have a high sequence similarity to *AP1*/*FRUITFULL* (*FUL*) of Arabidopsis. Following this study in diploid wheat, the *Vrn-1* genes of hexaploid wheat were identified as *WAP1* (*wheat AP1*) (Murai *et al.*, 2003; Trevaskis *et al.*, 2003) or *TaVRT-1* (*Triticum aestivum vegetative to reproductive transition-1*; Danyluk *et al.*, 2003). Transgenic and mutant analyses indicated that *VRN1* is a flowering promoter that has an indispensable role in the floral transition pathway of wheat (Murai *et al.*, 2003; Loukoianov *et al.*, 2005; Shitsukawa *et al.*, 2007a). In diploid wheat, the genes *Vrn-A*^*m*^*1* and *Vrn-A*^*m*^*2* are mainly responsible for the control of the vernalization response (Dubcovsky *et al.*, 1998). *Vrn-A*^*m*^*2* encodes a protein (ZCCT1) with the CO, CO-like and TOC1 (CCT) domain that was identified in the *CO*-like gene family, and named *VRN2* (Yan *et al.*, 2004). A transgenic study indicated that *VRN2* is a strong repressor of flowering in wheat. As *VRN2* is downregulated by vernalization, it is postulated that the VRN2 protein suppresses *VRN1* expression. The reduction of the VRN2 protein through vernalization allows *VRN1* transcription to increase gradually to enable flowering competence. Although large deletions within the first intron of the *VRN1* gene are associated with a spring habit (Fu *et al.*, 2005), there is currently no biochemical evidence that the VRN2 protein directly binds to the regulatory region of the *VRN1* gene. *TaVRT2*, a MADS-box gene with sequence similarity to Arabidopsis *SHORT VEGETATIVE PHASE* (*SVP*), has been identified as another flowering repressor in wheat (Kane *et al.*, 2005). TaVRT2 directly binds the CArG motif in the *VRN1* promoter, suggesting that TaVRT2 represses the transcription of *VRN1* in association with VRN2 (Kane *et al.*, 2007). Recently, another vernalization gene, *Vrn-3*, located on chromosome 7B, was shown to encode a homolog of Arabidopsis *FT*, and was named *VRN3* (Yan *et al.*, 2006). Analyses of transgenic plants showed that *VRN3* functions as a flowering promoter. These findings indicate that the genetic system that determines the vernalization response in wheat is quite different from that in Arabidopsis, a conclusion that is consistent with the absence of an *FLC*-like gene in the wheat genome.

The photoperiod (LD) response in wheat is determined by the dominant genes *Ppd-A1*, *Ppd-B1* and *Ppd-D1*, which control sensitivity to photoperiod, and are located on chromosomes 2A, 2B and 2D, respectively (Worland and Snape, 2001). Wheat is a quantitative LD plant, and short-day (SD) conditions delay the heading time. The *Ppd* gene acts to reduce the delay of heading under SD conditions (Murai *et al.*, 2003). Recently, a barley (*Hordeum vulgare*, 2n=2x=14, HH) ortholog of the *Ppd* genes, *Ppd-H1*, was identified as a pseudoresponse regulator (*PRR*) that showed most similarity to Arabidopsis *PRR7* (Turner *et al.*, 2005). In Arabidopsis, the PRR family consists of five members (PRR9, PRR7, PRR5, PRR3 and PRR1/TOC1) that are involved in circadian clock function (Mizuno and Nakamichi, 2005). This suggests that the barley *Ppd* gene is likely to be a circadian clock-associated gene. Comparative mapping indicated that the wheat *Ppd* gene is orthologous to the barley *Ppd* gene, a conclusion that has been supported by sequence and expression analyses (Beales *et al.*, 2007).

Narrow-sense earliness, which corresponds to the autonomous flowering pathway in Arabidopsis, refers to earliness in the flowering of fully-vernalized plants grown under LD conditions. Several quantitative trait loci (QTLs) have been identified in barley and wheat for this characteristic (Cockram *et al.*, 2007). GA accelerates the transition from the vegetative to reproductive phase in wheat in a similar manner as in Arabidopsis (Weibel, 1960; Pauli *et al.*, 1962). The wheat *SOC1* homolog, *wheat SOC1* (*WSOC1*), may function in the GA pathway (Shitsukawa *et al.*, 2007b).

In this study, we performed expression, mutant and transgenic studies to clarify the genetic network of flowering-time genes in wheat, especially the relationship of two flowering promoter genes, *VRN1* (an *AP1*/*FUL* homolog) and *VRN3* (an *FT* homolog). Expression analysis of a *VRN1* deletion mutant suggested that *VRN3* (*FT*) is upregulated by *VRN1* together with *CO* under LD conditions. Furthermore, our transgenic analysis of *VRN3* (*FT*) indicated that *VRN3* (*FT*) suppresses *VRN2* expression. Here, we present a model for the genetic network of flowering-time genes in wheat, in which *VRN1* is upstream of *VRN3* (*FT*), with a positive feedback loop through *VRN2*.

## Results

### Diurnal rhythmic expression patterns of *GI*, *CO* and *FT* in wheat leaves

**Figure 1:**
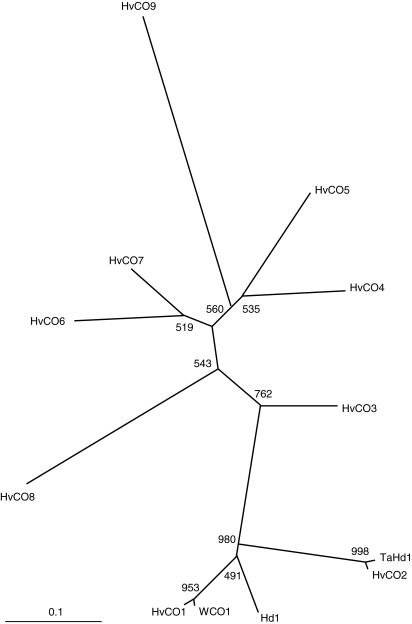
Phylogenetic tree of *CO*-like genes of wheat (*WCO1* and *TaHd1*), barley (*HvCO1*–*HvCO9*) and rice (*Hd1*). The phylogenetic tree was constructed by the neighbor-joining method using deduced amino acid sequences. The numbers at the nodes show bootstrap values after 1000 replicates.

Wheat *GI* (*TaGI1*) and wheat *FT* (*VRN3*) have been identified in previous work (Zhao *et al.*, 2005; Yan *et al.*, 2006). In this study, we identified wheat *CO* (*WCO1*) by using the PCR method with primers based on the sequence of barley *HvCO1* located on chromosome 7H (Griffiths *et al.*, 2003). In the barley genome, nine *CO* homologs have been identified. On the basis of sequence similarity and chromosomal synteny, *HvCO1* located on chromosome 7H may be the counterpart of rice *Hd1*. The mapping study showed that *WCO1* was located on homoeologous group 7 (7A, 7B and 7D) (Figure S1). Wheat chromosomes 7A, 7B and 7D are syntenic with rice chromosome 6 (the location of the *Hd1* gene), suggesting that *WCO1* is the counterpart of *Hd1*. It is known that *TaHd1*, previously identified as a wheat *CO*-like gene, is located on a group-6 homoeologous chromosome (Nemoto *et al.*, 2003), indicating that *TaHd1* is not an ortholog of *Hd1*. A phylogenetic tree of rice *Hd1* and barley *CO*-like genes, together with two wheat *CO*-like genes, *TaHd1* and *WCO1*, using deduced amino acid sequences (see Figure 1 for the tree, and Figure S2 for amino acid alignment), reinforced the conclusion that *WCO1* is the wheat ortholog of *Hd1/CO*. However, from their investigations with transgenic rice, Nemoto *et al.* (2003) reported that *TaHd1* has some function in flowering. In this study, therefore, both *WCO1* and *TaHd1* are used as wheat *CO* genes.

**Figure 2:**
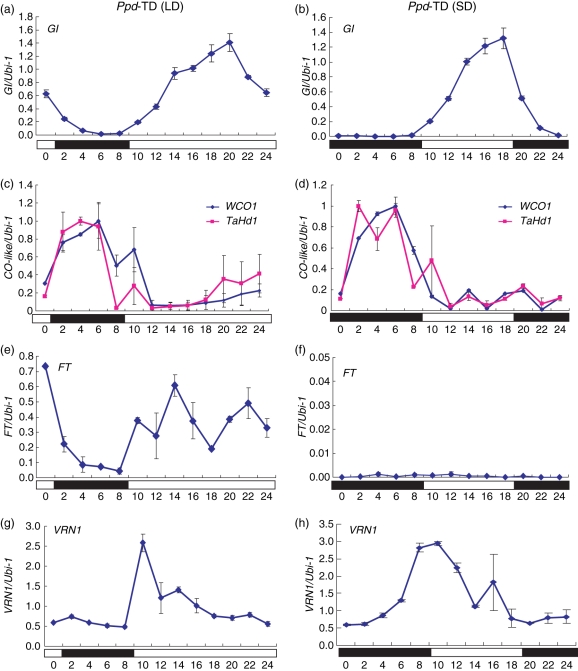
Diurnal expression patterns of *GI*, *CO*, *FT* and *VRN1* in spring wheat cv. Triple Dirk plants with *Ppd* dominant alleles (*Ppd*-TD), grown under long-day (LD) or short-day (SD) conditions. The expression patterns of *GI* (a and b), *CO* (*WCO1* and *TaHd1*) (c and d), *FT* (e and f) and *VRN1* (g and h) were analyzed by real-time PCR using TD plants at the three-leaf stage grown under LDs (16-h light/8-h dark) (a, c, e, g) or SDs (10-h light/14-h dark) (b, d, f, h) at 20°C. The *Ubiquitin* (*Ubi-1*) gene was used as an internal control for calculating the relative levels of *GI*, *CO* (*WCO1* and *TaHd1*), *FT* and *VRN1* genes. Each point represents the average of two replicates, and the error bars indicate the range. The white and black bars along the horizontal axes represent light and dark periods, respectively. The numbers on the horizontal axes indcate the time in hours.

The diurnal expression patterns of *GI* (*TaGI1*), *CO* (*WCO1* and *TaHd1*) and *FT* (*VRN3*) were examined in leaves at the three-leaf stage in spring wheat cv. Triple Dirk (TD) plants with *Ppd* dominant alleles (*Ppd*-TD), grown under LD or SD conditions (Figure 2). The expression analysis was performed using real-time PCR with primers that amplify all three homoeologs located on A, B and D wheat genomes. The shoot apical meristem (SAM) of the *Ppd*-TD plant transits from the vegetative to reproductive phase around at the four-leaf stage when grown under LD conditions. Under SD conditions the timing of the phase transition is a little delayed (occurring at the five-leaf stage). Under LD conditions, *GI* mRNA accumulated during the light period, and expression peaked late in the light phase (Figure 2a). *CO* (*WCO1* and *TaHd1*) mRNA accumulated during the dark period (Figure 2c), and *FT* mRNA accumulated from the beginning of the light phase (Figure 2e). Under SD conditions, *GI* mRNA expression showed a similar pattern as under LD conditions, although the peak of expression was shifted towards the end of the light period (Figure 2b). *CO* (*WCO1* and *TaHd1*) expression also showed a similar pattern as under LD conditions, but the *WCO1* expression was mostly confined to the dark period under SDs (Figure 2d). In contrast to *GI* and *CO*, no expression of *FT* was detected under SD conditions (Figure 2f). The expression analysis did not conflict with the idea that the functional hierarchy, *GI* → *CO* → *FT*, is conserved in wheat. Analysis of the diurnal expression pattern in barley also indicated conservation of this cascade (Turner *et al.*, 2005).

**Figure 3:**
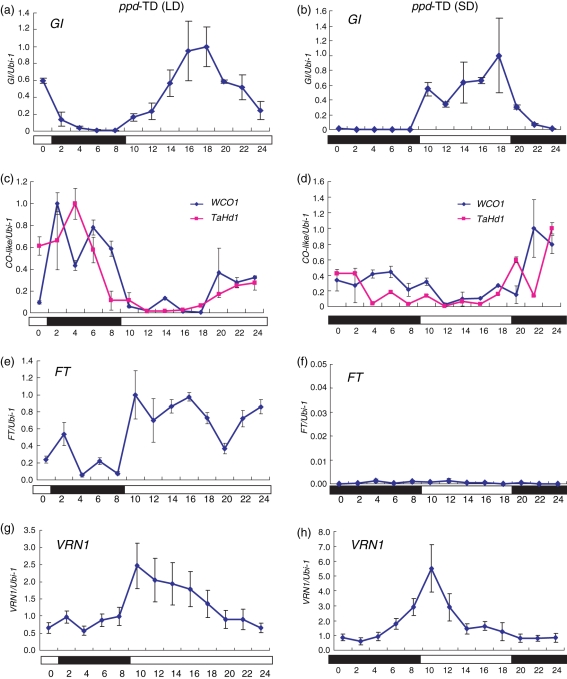
Diurnal expression patterns of *G1*, *CO*, *FT* and *VRN1* in spring wheat cv. Triple Dirk (TD) plants with *ppd* recessive alleles (*ppd*-TD), grown under long-day (LD) or short-day (SD) conditions. The expression patterns of *GI* (a and b), *CO* (*WCO1* and *TaHd1*) (c and d), *FT* (e and f) and *VRN1* (g and h) were analyzed by real-time PCR using TD plants at the three-leaf stage grown under LDs (16-h light/8-h dark) (a, c, e, g) or SDs (10-h light/14-h dark) (b, d, f, h) at 20°C. The *Ubiquitin* (*Ubi-1*) gene was used as an internal control for calculating the relative levels of *GI*, *CO* (*WCO1* and *TaHd1*), *FT* and *VRN1* genes. Each point represents the average of two replicates, and the error bars indicate the range. The white and black bars along the horizontal axes represent light and dark periods, respectively. The numbers on the horizontal axes indicate the time in hours.

The diurnal expression patterns of *GI*, *CO* and *FT* were also examined in leaves at the three-leaf stage in TD plants with *ppd* recessive alleles (*ppd*-TD), grown under LD or SD conditions (Figure 3). The SAM of the *ppd*-TD plant transits from the vegetative to the reproductive phase around at the four-leaf stage when grown under LD conditions, whereas the timing of the phase transition is very delayed (occurring at the six-leaf stage) under SD conditions. The expression of these genes in *ppd*-TD showed similar patterns as in *Ppd*-TD plants, except for the expression patterns of *GI* and *CO* genes under SD conditions (Figure 3). In *GI*, *ppd*-TD plants showed a dual peak expression pattern in the light period (Figure 3b). Furthermore, in both *WCO1* and *TaHd1*, no peaks were observed late in the dark period compared with the expression patterns in *Ppd*-TD plants (Figure 3d). As the *Ppd* gene should be one of the circadian clock component genes, the results indicate that the *GI* and *CO* genes are circadian clock-regulated genes. The difference in the timing of the phase transition between *Ppd*-TD and *ppd*-TD under SD conditions may be associated with the difference of diurnal expression patterns of *GI* and *CO* genes.

### *VRN1* also shows a diurnal expression pattern in wheat leaves

At the three-leaf stage, spring wheat *Ppd*-TD plants showed a diurnal pattern of *VRN1* expression under both LD (Figure 2g) and SD (Figure 2h) conditions. *VRN1* expression peaked at the beginning of the light period under both LDs and SDs, suggesting that the expression of *VRN1* is regulated by the light–dark cycle. Comparison of the expression patterns of *VRN1* and *FT* under LD conditions (Figure 2e, g) showed that the accumulation of *FT* transcripts in the light period followed the upregulation of *VRN1*. This suggests that the *VRN1* expression is associated with the *FT* expression in wheat leaves under LD conditions. Although *VRN1* was expressed under SD conditions, no *FT* expression was detected (Figure 2f, h). In *ppd*-TD plants, *VRN1* showed a similar expression pattern as in *Ppd*-TD: the expression peaked at the beginning of the light period under both LD (Figure 3g) and SD (Figure 3h) conditions. This result supports the idea that the expression of *VRN1* is regulated by the light–dark cycle. As in *Ppd*-TD, *ppd*-TD plants showed that the accumulation of *FT* transcripts in the light period followed the upregulation of *VRN1*, suggesting that the *VRN1* expression is associated with *FT* expression under LD conditions.

### *FT* is not expressed in *mvp*, a deletion mutant of *VRN1*

**Figure 4:**
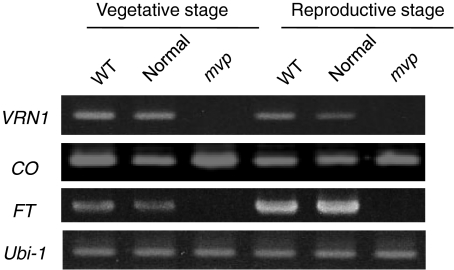
Expression patterns of *VRN1*, *CO* (*WCO1*) and *FT* in the *mvp* mutant, together with the corresponding wild type (WT) and normal lines. The normal line comprises M_3_ plants with a normal phenotype that segregated from an *mvp* heterozygous M_2_ plant. The reproductive stage was defined here as the growth stage when the internodes of WT and normal plants were elongating. At this stage *mvp* mutants are still in the vegetative phase. Gene expression levels were compared at the exponential phase of RT-PCR amplification. The *Ubiquitin* (*Ubi-1*) gene was used as an internal control.

The einkorn wheat mutant, *maintained vegetative phase* (*mvp*), was induced by accelerated nitrogen ion irradiation treatment, and was identified by its inability to transit from the vegetative to reproductive phase (Shitsukawa *et al.*, 2007a). The *mvp* mutant cannot transit to the reproductive phase under any growth conditions (LD, SD, non-vernalized and vernalized). In the previous study, genetic analysis indicated that the *mvp* phenotype is controlled by one locus, and we demonstrated that the *mvp* mutation was caused by the deletion of the *VRN1* coding and promoter regions (Shitsukawa *et al.*, 2007a). The *mvp* mutant, *mvp-1*, used in this study was obtained from einkorn spring wheat strain KU104-2, with dominant alleles of *VRN1* and null alleles of *VRN2* (Figure S3). Yan *et al.* (2004) revealed that spring einkorn wheats are classified into three types in the *VRN2* allele: wild type, R/W mutant type and deletion type. KU104-2 is a deletion type spring einkorn wheat (Figure S3). Consequently, the *mvp* mutant has deletion alleles of both *VRN1* and *VRN2*, because it was derived from KU104-2. The expression patterns of *VRN1*, *CO* (*WCO1*) and *FT* were analyzed by RT-PCR in *mvp*, the corresponding wild type (WT) and the normal lines, using autumn-sown plants in the experimental field (Figure 4). The normal line comprised M_3_ plants with a normal phenotype that segregated from an *mvp* heterozygous M_2_ plant. *VRN1* gene expression was observed in WT and normal lines at both vegetative and reproductive growth stages in spring to summer, but was not detected in the *mvp* mutants (Shitsukawa *et al.*, 2007a). The reproductive stage is defined here as the growth stage in which the internodes of WT and normal plants elongate. At this stage, the *mvp* mutants are still in the vegetative phase. In contrast to *VRN1*, the expression of *CO* was detected in *mvp* mutants, indicating that *CO* acts upstream of or in a different pathway to *VRN1*. No expression of *FT* was observed in the *mvp* mutants. Because the *mvp* mutant has intact coding and promoter regions of the *FT* gene in the genome (Figure S4), the present observation indicates that no expression of *FT* resulted from the null allele of *VRN1*.

**Figure 5:**
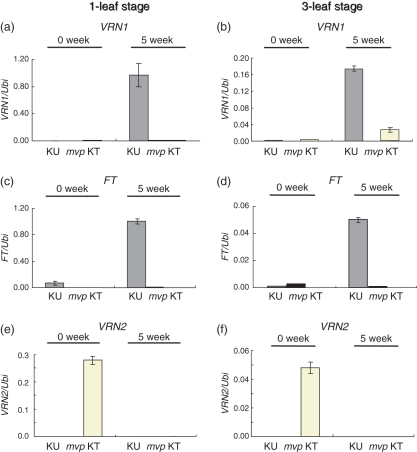
The effect of vernalization on the expression levels of *VRN1*, *FT* and *VRN2* in *mvp* mutant, together with WT spring strain KU104-2 (KU) and winter strain KT10-1 (KT) of einkorn wheats. The *mvp* mutant was derived from KU by ion-beam irradiation. The expression levels of *VRN1* (a and b), *FT* (c and d) and *VRN2* (e and f) were determined by real-time PCR. Seedlings at the one-leaf stage (a, c, d) or the three-leaf stage (b, d, f) grown under long-day (LD; 16-h light/8-h dark) conditions were transferred into a cold chamber at 4°C for 5 weeks. Leaves were sampled before and after vernalization treatment, and were used for analysis. The averages of two replicates are shown, together with error bars indicating the range. The *Ubiquitin* (*Ubi*) gene was used as an internal control for calculating the relative levels of expression of the *VRN1*, *FT* and *VRN2* genes.

It has been reported that the expression of *FT* increased significantly in wheat leaves in response to vernalization, just as the expression of *VRN1* did (Yan *et al.*, 2006). To examine the effect of vernalization on gene expression patterns of *VRN1*, *FT* and *VRN2* in the *mvp* mutant together with the WT, seedlings at the one-leaf stage or at the three-leaf stage were cold-treated, and gene expression levels were analyzed by real-time PCR (Figure 5). At these stages, the SAMs of both the *mvp* mutant and the WT plants are still in the vegetative phase. In strain KU104-2 plants at the one-leaf and three-leaf stages, *VRN1* and *FT* were clearly upregulated by cold treatment (Figure 5a–d), but no *VRN2* expression was observed because of the lack of *VRN2* alleles (Figure 5e,f). KU104-2 is an early-heading type, and high expression of *FT* suggests that it has the dominant allele of *FT*. In the *mvp* mutant, no expression of *FT* as well as of *VRN1* was observed at the one-leaf and three-leaf stages (Figure 5a-d), confirming the results obtained from the field-grown plants (Figure 4). We also examined normal winter einkorn wheat strain KT10-1 with recessive *vrn1* and dominant *VRN2*. KT10-1 is a late-heading type, with no expression of *FT* at the one-leaf and three-leaf stages (Figure 5c,d). At the one-leaf stage of KT10-1 plants, the *VRN2* expression level was high in non-vernalized plants, and was dramatically decreased in vernalized plants (Figure 5e). At the three-leaf stage, the *VRN2* expression was still at a high level in the non-vernalized plants, and was downregulated by cold treatment (Figure 5f), whereas the *VRN1* expression was upregulated (Figure 5b). Contrary to the spring strain KU104-2 and the *mvp* mutant, the winter strain KT10-1 showed a high expression level of *VRN2* at the three-leaf stage (Figure 5f), suggesting that the expression of *VRN2* was not affected by aging from the one-leaf to the three-leaf stage in the winter strain.

### Overexpression of *FT* induces the downregulation of *VRN2* and the upregulation of *VRN1*

**Figure 6:**
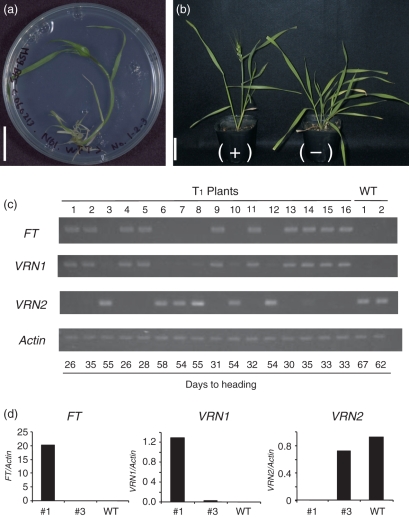
Analysis of phenotype and gene expression in transgenic wheat transformed with a *35S:VRN3-D* construct. (a) A spikelet emerged from a transgenic T_0_ plant grown in a petri dish at the three-leaf stage under short-day (SD; 8-h light/16-h dark) conditions at 25°C. Scale bar: 2 cm. (b) Transgenic T_1_ plants derived from a T_0_ plant showing segregation for the presence (+) or absence (−) of the transgene. The T_1_-positive plant (left) showed early heading. The T_1_ (+) and (−) plants illustrated here correspond to plants 1 and 3 in (c), respectively. The plants were grown under SDs (10-h light/14-h dark) at 20°C, and were photographed at 64 days after sowing. Scale bar: 5 cm. (c) The expression of *FT*, *VRN1* and *VRN2* in T_1_ plants segregating for the *FT* transgene, and in non-transformants (i.e. wild type, WT). RT-PCR analysis was performed using the seedlings at the one-leaf stage. Gene expression levels were compared at the exponential phase of RT-PCR amplification. The *Actin* gene was used as an internal control. Numbers indicate the days to heading in each T_1_ plant. The plants were grown under SDs (10-h light/14-h dark) at 20°C. (d) Expression levels of *FT*, *VRN1* and *VRN2* in transformants 1 (positive) and 3 (negative), and non-transformant (WT) determined by real-time PCR analysis. The mRNA levels were normalized against the mRNA level of *Actin*.

Using spring wheat cv. Norin 61 (N61), we developed transgenic plants with *VRN3-D* cDNA driven by *P35S*. *VRN3-D* is a homoeolog of wheat *FT* (*VRN3*) located on the D genome. In culture in a petri dish, a transgenic T_0_ plant with overexpression of *FT* showed extremely early heading (Figure 6a), indicating that *FT* is a strong activator of the flowering pathway. Transgenic wheat plants were grown in a growth chamber under SDs. mRNAs were extracted from leaves of non-vernalized seedlings at the one-leaf stage, and the expression levels of *FT*, *VRN1* and *VRN2* were examined. At this stage, the SAMs of transgenic and non-transgenic (WT) wheat are still in the vegetative phase. In WT plants, the expression of *FT* and *VRN1* were not observed, whereas a high expression of *VRN2* was detected (Figure 6c). Overexpression of *FT* was observed in 10 T_1_ plants, 1, 2, 4, 5, 9, 11 and 13–16, which showed an early heading trait compared with non-transgenic plants (Figure 6b, c). To investigate whether the RT-PCR patterns of *FT* showed expression profiles of the *VRN3-D* transgene, the amplified RT-PCR products of positive transgenic lines 4 and 5 were cloned and sequenced. The sequences of RT-PCR products were identical with those of *VRN3-D*, and differed from the sequences of *VRN3-A* and *VRN3-B* (Figure S5), indicating that the RT-PCR analysis of *FT* exhibited the expression profile of the *VRN3-D* transgene.

The positive transgenic T_1_ plants showed a reduction in *VRN2* mRNA levels, but WT plants and T_1_ null segregants (3, 6, 7, 8, 10 and 12) exhibited a high expression of *VRN2* (Figure 6c). Furthermore, the T_1_ plants with overexpressing *FT* showed a high expression of *VRN1*. These findings suggest that the downregulation of *VRN2* is associated with the upregulation of *FT* in the positive T_1_ plants, and the upregulation of *VRN1* in the positive T_1_ plants probably resulted from the downregulation of *VRN2*. Real-time PCR analysis confirmed that the positive transgenic plant 1 with high expression of *FT* also showed a downregulation of *VRN2* and an upregulation of *VRN1*, whereas the negative transgenic plant 3 and the WT showed a high level of *VRN2* transcripts and a low level of *VRN1* transcripts (Figure 6d). In the previous transgenic studies of wheat *FT* (*VRN3*) (Yan *et al.*, 2006; Li and Dubcovsky, 2008), it has also been shown that *VRN1* was upregulated in transgenic plants. However, the previous study did not examine the expression of *VRN2*.

## Discussion

By analyzing the expression patterns of flowering genes (*VRN1*, *VRN2* and *FT*) in the *mvp* mutant with null alleles of both *VRN1* and *VRN2*, and transgenic plants with overexpressing *FT*, we hypothesized that *VRN1* acts upstream of *FT*, and upregulates *FT* with a positive feedback loop through *VRN2* in wheat leaves. This genetic network among *VRN1*, *VRN2* and *FT* is different from the two other models previously presented (Hemming *et al.*, 2008; Li and Dubcovsky, 2008). In this study, analysis of the *mvp* mutant provided crucial information on the relationship between *VRN1* and *FT*. The other two models can not explain the present results: no expression of *FT* was observed in the *mvp* mutant with null alleles of both *VRN1* and *VRN2*.

### *VRN1* is upstream of *FT* in the wheat flowering-gene network

The present study indicated that wheat *FT* (*VRN3*) was upregulated by vernalization in a similar fashion to *VRN1* (Figure 5a–d), as was also reported in a previous study (Yan *et al.*, 2006). This fact suggests that *FT* as well as *VRN1* is associated with the vernalization pathway in wheat leaves. What is the relationship between *VRN1* and *FT*? Recently, we described an einkorn wheat mutant, *mvp*, obtained by ion-beam treatment, that does not transit from the vegetative to reproductive phase (Shitsukawa *et al.*, 2007a). The *mvp* mutant was caused by the deletion of *VRN1*. This clearly demonstrated that *VRN1* is an indispensable gene for phase transition in wheat. No expression of *FT* was observed in the *mvp* mutant (Figures 4 and 5c,d), suggesting that expression of *FT* is related to the expression of *VRN1*. In this study, we also demonstrated that *VRN1* shows a diurnal expression pattern under both LD (Figures 2g and 3g) and SD (Figures 2h and 3h) conditions. Under LD, an accumulation of *FT* transcripts follows the upregulation of *VRN1* (Figures 2e,g and 3e,g). Based on these findings, we hypothesize that *VRN1* acts upstream of *FT*, and activates *FT* expression under LD conditions. This putative genetic interaction would not be completely unexpected, because in Arabidopsis a MADS-box protein, FLC, interacts directly with *FT* sequences, and regulates *FT* expression as part of a protein complex (Helliwell *et al.*, 2006). Under SD conditions, *VRN1* is expressed in a similar pattern as under LDs (Figure 2h and 3h), but *FT* is not expressed (Figures 2f and 3f). This suggests that the upregulation of *FT* by *VRN1* involves other factor(s).

In Arabidopsis, it was demonstrated that *FT* is expressed in the vascular tissue of leaves, and that the FT protein moves from the leaves to the SAMs as a long-distance signal for flowering induction (Corbesier *et al.*, 2007; Jaeger and Wigge, 2007; Mathieu *et al.*, 2007). The FT protein has also been shown to function as a florigen in rice (Tamaki *et al.*, 2007) and cucurbits (Lin *et al.*, 2007). Although it is not known if the FT protein has a florigenic effect in wheat, our transgenic study demonstrated that *FT* is a strong activator of flowering in this species (Figure 6a–c). This conclusion is supported by the results of another transgenic study (Yan *et al.*, 2006). Arabidopsis shows an upregulation of *FT* under LDs, and a downregulation under SDs, in which the CO protein induces the expression of *FT* in a light-dependent manner at dusk (Yanovsky and Kay, 2003). Recently, the abundance of CO protein is regulated by the ubiquitin-mediated proteolysis in darkness (Jang *et al.*, 2008; Liu *et al.*, 2008). Based on these studies, we can postulate that functionally stable CO proteins (WCO1 and/or TaHd1) could be candidates for the co-factors in wheat flowering.

The FT protein interacts with the bZIP transcription factor FD at the SAM, and activates the floral meristem identity gene *AP1* in Arabidopsis (Abe *et al.*, 2005; Wigge *et al.*, 2005). Recently, a wheat ortholog of *FD* (*TaFDL2*) was identified (Li and Dubcovsky, 2008). *TaFDL2* is expressed in wheat leaves together with *VRN1*, and TaFDL2 protein can interact with FT protein and binds *in vitro* with the promoter region of *VRN1*. Based on these results, a hypothesis that *VRN1* is upregulated by *FT* in wheat leaves was presented (Li and Dubcovsky, 2008). However, *TaFDL2* is expressed in SAMs as well as in leaves (Li and Dubcovsky, 2008), and *VRN1* is also expressed in SAMs (Preston and Kellogg, 2008). Therefore, it is possible that the FT-TaFDL2-VRN1 complex functions in SAMs rather than in leaves, as in Arabidopsis. The expression analysis supported the idea that *VRN1* has discrete roles of flowering competency in leaves and of floral meristem identity in SAMs (Preston and Kellogg, 2008).

### *VRN1* and *FT* are upregulated by cold, regardless of *VRN2*

Based on the reciprocal expression patterns of *VRN2* and *VRN1* when plants are vernalized, and the knowledge of the epistatic nature that *VRN2* has on *VRN1*, a model of the vernalization pathway has been proposed (Yan *et al.*, 2003, 2004, 2006). In this model, *VRN2* encodes a repressor of *VRN1*, and, as the vernalization process reduces the abundance of the *VRN2* gene products, *VRN1* transcription gradually increases, leading to competency in flowering. In this study, we demonstrated that *VRN1* is upregulated by cold in the spring einkorn wheat strain KU104-2 that has null alleles of *VRN2* (Figure 5a,b). This clearly indicates that the expression of *VRN1* is regulated by cold, regardless of *VRN2*. This is also supported by the observation that in barley a cold signal induces *VRN1* expression, but not through the repression of *VRN2* (Trevaskis *et al.*, 2006, 2007; Hemming *et al.*, 2008). This idea is based on the finding that *VRN2* expression is not affected by vernalization under SD conditions (Trevaskis *et al.*, 2006). Furthermore, in barley doubled haploid (DH) lines that lack the *VRN2* locus, vernalization can still induce *VRN1* and accelerate flowering (Hemming *et al.*, 2008). These findings suggest an additional pathway in which *VRN1* is upregulated by vernalization independently of *VRN2*.

To explain the relationship between *VRN1* and *FT*, two different models were previously presented. In the first model, both *FT* and *VRN1* are suppressed by *VRN2*. Vernalization downregulates *VRN2*, leading to the upregulation of both *FT* and *VRN1* (Yan *et al.*, 2006). In this model, a long photoperiod induces *FT* expression, and then *FT* upregulates *VRN1*. However, this model cannot explain why KU104-2 lacking *VRN2* shows an upregulation of *FT* and *VRN1* by vernalization (Figure 5a–d). Furthermore, this model cannot explain why the *mvp* mutant lacking both *VRN1* and *VRN2* shows no expression of *FT* (Figure 5c,d). The second model indicated that *FT* is suppressed by *VRN2*. Vernalization upregulates *VRN1* and *VRN1* downregulates *VRN2*, leading to the upregulation of *FT* (Hemming *et al.*, 2008). In this model, a long photoperiod induces *FT* expression. This model can explain why the cold signal upregulates both *VRN1* and *FT*, but does not explain why KU104-2 lacking *VRN2* shows an upregulation of *FT* by vernalization (Figure 5c,d), nor why the *mvp* mutant lacking both *VRN1* and *VRN2* shows no expression of *FT* (Figure 5c,d).

### *FT*, a flowering activator, suppresses the expression of *VRN2*

In agreement with Yan *et al.* (2006), we found that transgenic plants overexpressing *FT* headed early (Figure 6a–c), indicating that *FT* is an activator of the flowering pathway in wheat. The early-heading transgenic plants with high *FT* expression showed an upregulation of *VRN1* and a downregulation of *VRN2*, compared with control non-transformants and null segregants (Figures 6c,d). Assuming that the upregulation of *VRN1* resulted from the downregulation of *VRN2* (Yan *et al.*, 2003, 2004), our results suggest that *VRN2* is downregulated by *FT* in the transgenic plants.

An alternative model in which *VRN2* downregulates *FT* was presented in barley, based on the results showing that transgenic barley plants overexpressing *VRN2* showed no expression of *FT* (Hemming *et al.*, 2008). However, this observation does not conflict with our model, because it is possible that the overexpression of *VRN2* induces the downregulation of *VRN1*, and that the low level of *VRN1* expression leads to a decrease in the expression of *FT*. In the previous study of *VRN2* transgenic barley, Hemming *et al.* (2008) reported that there was no difference of *VRN1* expression level between transgenic plants and null segregants. However, the previous study took no account of the diurnal expression pattern of *VRN1*, and so the expression level of *VRN1* could not be accurate. More precise work is necessary to investigate the alternative model.

Feedback regulatory loop models have been developed based on studies using an F_2_ population of diploid wheat (Loukoianov *et al.*, 2005), or a DH population of barley (Trevaskis *et al.*, 2006), segregating *VRN1* and *VRN2* alleles. In these models, *VRN2* acts as a repressor of flowering, and the expression of *VRN2* is directly or indirectly repressed after the initiation of *VRN1* expression. Our model proposes that *VRN2* expression is controlled by *VRN1* through *FT*, because *FT* should be upregulated by *VRN1*, and *VRN2* should be downregulated by *FT*.

### The *VRN1–FT–VRN2* triangle model for flowering in wheat

**Figure 7:**
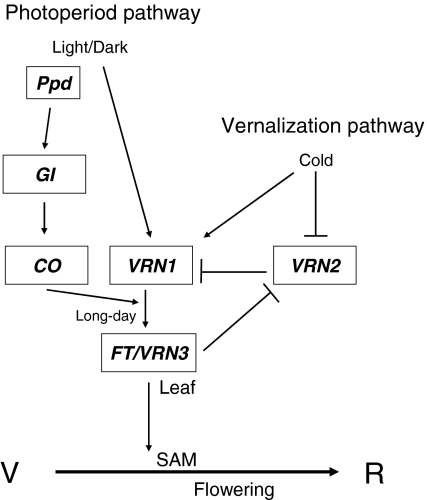
A model for the interaction of flowering-time genes in wheat. This model includes the assumption that *VRN2* suppresses *VRN1*, and that a *GI* → *CO* → *FT* cascade functions in the photoperiod pathway for wheat flowering. Furthermore, *Ppd* is supposed to function in the circadian clock system, and upstream of *GI* and *CO*. This triangle model of *VRN1–FT–VRN2* for the regulation of the phase transition from vegetative (V) to reproductive (R) growth in wheat, based on the results of expression, mutant and transgenic studies, was presented in this study. We propose that *FT* plays an integrative role in both the *CO*-related photoperiod pathway and the *VRN1*-related vernalization pathway. The proposed upregulation of *FT* by *VRN1* is based on the analysis of the *mvp* mutant, and the downregulation of *VRN2* by *FT* is based on the transgenic study. Note that the interaction of these genes occurs in leaves. Arrows represent promotive interactions, and T-bars represent inhibitory interactions.

According to the present results, one possible model for the regulation of the floral transition in wheat is shown in Figure 7. This model includes the assumption that *VRN2* suppresses the expression of *VRN1*, and that a *GI* → *CO* →*FT* cascade functions in the photoperiod pathway in wheat flowering. Furthermore, *Ppd* is supposed to function in the circadian clock system, and upstream of *GI* and *CO*. Our expression and mutant analyses suggested that *VRN1* acts upstream of *FT*, and possibly acts with *CO* (*WCO1* and/or *TaHd1*) to activate *FT* expression under LD conditions. Vernalization downregulates *VRN2* and upregulates *VRN1*, independently of each other. Furthermore, the transgenic study suggested that *VRN2* is downregulated by *FT*. Based on these results, a feedback triangle of *VRN1*–*FT*–*VRN2* is suggested to be involved in wheat flowering. Our model can explain why *VRN1* represses *VRN2* expression (Loukoianov *et al.*, 2005; Trevaskis *et al.*, 2006). However, our model does not describe why *VRN2* is downregulated by a short photoperiod (Dubcovsky *et al.*, 2006; Trevaskis *et al.*, 2006). This might be caused other photoperiod pathway functions under SD conditions. Note that the gene interactions shown in Figure 7 are events that occur in leaves. As in Arabidopsis and rice, FT proteins could be the florigen that moves from the leaves into the SAMs to determine floral meristem identity in wheat.

The Arabidopsis genome contains three *AP1*-like genes, *AP1*, *CAULIFLOWER* (*CAL*) and *FUL*, all of which act redundantly at the SAM (Ferrandiz *et al.*, 2000). The grass family, including wheat, has two paralogues of *AP1/FUL* in the genome, *FUL1/VRN1* and *FUL2*, both derived from the *FUL* lineage (Preston and Kellogg, 2007). Expression analyses indicated that both *VRN1* and *FUL2* are expressed in SAMs as well as in leaves (Preston and Kellogg, 2008). The upregulation of *VRN1* is significantly later in SAMs than in the leaves of vernalized plants, suggesting that *VRN1* may perform discrete roles in leaves and in SAMs. This idea is highly consistent with our model in which *VRN1* functions in leaves to obtain flowering competency, and acts in SAMs to facilitate the transition to flowering. It has been reported that the expression of *FUL2* increased following the attainment of flowering competency (Preston and Kellogg, 2008), suggesting that *FUL2* functions redundantly in SAMs for the transition to flowering, together with *VRN1*.

In diploid wheat, an epistatic interaction between *VRN1* and *VRN2* has been demonstrated (Tranquilli and Dubcovsky, 2000). In contrast to *VRN1*, which is a dominant allele for spring growth habit, the *VRN2* allele is dominant for winter growth habit. The effect of *VRN1* on heading time is significant only when the dominant *VRN2* allele is present, that is, *VRN2* is epistatic to *VRN1*. Our model can explain the epistatic relationship between *VRN1* and *VRN2*. In plants with *vrn2* recessive alleles, high expression of *VRN1* induces *FT* regardless of which *VRN1* alleles are present. Thus, in the absence of VRN2 activity, the LD induction of *FT* causes early flowering, which overcomes the requirement for vernalization (spring habit). This is in good agreement with the observation that *FT* is expressed at higher levels in einkorn wheat lines that lack *VRN2* (Yan *et al.*, 2006; Figure 5c,d in this study). In plants with *VRN2* dominant alleles, *VRN1* expression is high in the presence of dominant *VRN1* alleles because *VRN2* cannot suppress the expression of *VRN1*. The expression of *VRN2* is then downregulated by *FT*, inducing flowering without vernalization (spring habit). In the presence of recessive *vrn1* alleles, *VRN1* expression is suppressed by *VRN2* under non-vernalizing conditions. Vernalization decreases *VRN2* expression and induces *VRN1* expression, thereby inducing flowering (winter habit). Once *VRN1* is upregulated, *FT* is induced and *VRN2* is suppressed.

We conclude that *FT* is the principal factor that integrates the *CO-*related photoperiod and *VRN1*-related vernalization pathways in wheat leaves, leading to the competence of flowering in wheat plants. As flowering is a very complicated characteristic in wheat, as well as in Arabidopsis, the present model of wheat flowering will undoubtedly need to be modified in the future following the cloning and analysis of novel genes.

## Experimental procedures

### Plant materials

Bread wheat (*T. aestivum*, 2n=6x=42, genome constitution AABBDD) cv. Chinese Spring (CS) was used for the cDNA cloning for *WCO1*. CS is a spring wheat cultivar, and is known to carry the vernalization-insensitive (spring habit) gene, *Vrn-D1.* Spring wheat cv. Triple Dirk (TD) with *Ppd* dominant alleles (*Ppd*-TD) or *ppd* recessive alleles (*ppd*-TD) were used for the diurnal expression studies in a growth chamber. *Ppd*-TD is not completely photoperiod-insensitive, and SD conditions delay the heading time compared with LD conditions (Murai *et al.*, 2003). *ppd*-TD shows early heading as much as *Ppd*-TD under LD conditions, but shows extremely late heading under SD conditions (Murai *et al.*, 2003). Both *Ppd*-TD and *ppd*-TD carry the vernalization-insensitive (spring habit) genes *Vrn-A1* and *Vrn-B1*. Spring wheat cv. N61, which carries *Vrn-D1*, was used for the transgenic study.

The einkorn wheat (*T. monococcum*, 2n=2x=14, A^m^A^m^) mutant, *mvp*, induced by nitrogen ion-beam treatment (Shitsukawa *et al.*, 2007a), was used for expression analyses. The *mvp* mutant was identified by its inability to transit from the vegetative to reproductive phase under any environmental conditions. The *mvp* mutation was caused by deletion of the *VRN1* promoter and coding regions. The *mvp* mutant, *mvp-1*, used in this study was obtained from spring einkorn wheat strain KU104-2 with dominant alleles of *VRN1* and null alleles of *VRN2*. Spring einkorn wheats are classified into three types in the *VRN2* allele: WT, R/W mutant type and deletion type (Yan *et al.*, 2004). KU104-2 is a deletion type spring einkorn wheat (Figure S3). Consequently, the *mvp* mutant has deletion alleles of both *VRN1* and *VRN2*, because it was derived from KU104-2.The *mvp* mutant and original WT strain, together with winter einkorn wheat strain KT10-1, with recessive *vrn1* and dominant *VRN2*, were used in the expression study.

### Growth conditions

For the diurnal expression study, non-vernalized plants were grown under LD (16-h light/8-h dark) or SD (10-h light/14-h dark) conditions at 20°C (100 μE m^−2^ s^−1^). The mutant study was performed using autumn-sown plants in the experimental field. For the vernalization study of the mutant, seedlings at the one- or three-leaf stage that had been grown in a growth chamber under LD conditions were transferred into a cold chamber at 4°C (20 μE m^−2^ s^−1^) for 0 or 5 weeks, and were then used for expression analysis. Transgenic plants were grown in a growth chamber under SD conditions at 20°C (100 μE m^−2^ s^−1^) for 5 weeks.

### Cloning and phylogenetic analysis of *WCO1*

The full-length cDNA sequence of *WCO1* was amplified with PCR primers designed using the sequence of barley *HvCO1* (Griffiths *et al.*, 2003): WCO1-full-3L (5′-TGCATGGTCTTTGTGGTG-3′) and WCO1-full-3R (5′-ATCCAACCATTATTCAGAGCAT-3′). Sequence data of *WCO1* are found in the DDBJ/EMBL/GenBank data libraries under the accession number AB361064. The chromosomal location of *WCO1* was determined by PCR analysis using nulli-tetrasomic CS lines with homoeolog-specific primers (Figure S1). A phylogenetic analysis was conducted using the neighbor-joining method with the deduced amino acid sequence (Figure S2). The accession numbers of other genes in the phylogenetic tree are as follows: *Hd1* (AB041838); *TaHd1* (AB094490); *HvCO1* (AF490468); *HvCO2* (AF490469); *HvCO3* (AF490471); *HvCO4* (AF490475); *HvCO5* (AY082958); *HvCO6* (AY082961); *HvCO7* (AY082963); *HvCO8* (AY082964); and *HvCO9* (AY082965).

### Sequence analysis of *VRN3* in *mvp*

PCR analyses of genomic DNA from WT (KU104-2) einkorn wheat and *mvp* plants were performed with PCR primers designed using the sequence of *VRN3* (DQ890162): for the promoter region, CArG1-Fw (5′-GCTTTTTTCCTAATACGGCCCGCGTC-3′) and CArG3-Re (5′-CACTTTATATAGGGCCGAAAAG-3′); for the coding region, CArG3-Fw (5′-CTTTTCGGCCCTATATAAAGTG-3′) and CArG4-Re (5′-GTGTTGCCAATATATAGGTAATGC-3′). The amplified PCR products were sequenced (Figure S4).

### RT-PCR analysis

Total RNAs were extracted from leaves using ISOGEN (Nippon Gene, http://www.nippongene.com), and cDNAs were synthesized from the total RNAs with oligo dT primer in accordance with the protocol for the Ready-To-Go T-Primed First-Strand Kit (GE Healthcare, http://www.gehealthcare.com). Leaves were sampled from the plants 2 h after the start of the light period. The RT-PCR analysis of einkorn wheat was performed using gene-specific primer sets for *VRN1* (VRN1-BAC 81655L and VRN1-BAC 82017R), *WCO1* (WCO1-L and WCO1-R) and *FT* (WFT-all-3L and WFT-all-3R). The Ubiquitin gene was used as the control with the primer set Ubi-1L and Ubi-1R. For the expression analysis of transgenic plants, total RNA was extracted from the first leaf of each plant using a Get Pure RNA Kit (Dojindo Laboratories, http://www.dojindo.com). Leaves were sampled from the plants 2 h after the start of the light period. RT-PCR was performed using the RNA PCR Kit (AMV) Version 2.1 (Takara Bio, http://www.takara-bio.com) with AmpliTaq Gold DNA Polymerase (Applied Biosystems, http://www.appliedbiosystems.com). Gene-specific primer sets were used for *FT* (WFT-FW and WFT-RV), *VRN1* (WAP1-556L and WAP1-982R) and *VRN2* (VRN2-FW2 and VRN2-RV2). Wheat actin gene (*Actin*) was used as the control with the primer set ACT-FW and ACT-RV. The sequences of the primer sets and the annealing temperatures are shown in Table S1. In all experiments, the RT-PCR analyses were performed in the exponential range of amplification. To determine the sequence of RT-PCR products of *VRN3* amplified from transgenic plants, RT-PCR products from positive transgenic lines 4 and 5 were directly sequenced (Figure S5).

### Real-time PCR analysis

In the diurnal expression study and mutant analysis, real-time PCR analyses were performed using a LightCycler 2.0 (Roche Diagnostics, http://www.roche.com/diagnostics) with gene-specific primer sets for *GI* (TaGI-3L and TaGI-3R), *TaHd1* (TaHd1-2L and TaHd1-2R), *FT* (WFT-F4 and WFT-R4), *VRN1* (WAP1-545L and WAP1-698R) and *VRN2* (ZCCT1-1Lt and ZCCT1-1Rt). The PCR primers and annealing temperatures for *WCO1* were the same as in the RT-PCR study. The quantity of transcripts was determined by the SYBR Green fluorescence of *Actin* using the primer set actin361-L and actin361-R. The *Ubi-1* gene was also used as the internal control using the same primer set as in the RT-PCR study. In the transgenic study, real-time PCR was carried out using the Stratagene MX3000 Real-Time PCR system (Stratagene, http://www.stratagene.com/). The template cDNAs were amplified with Brilliant SYBR Green QPCR Master Mix using the same primer sets for *WFT*, *WAP1* and *VRN2* as in the RT-PCR experiment, at and annealing temperature of 60°C. The data were analyzed using Stratagene MXPRO ver. 3.0 software (Stratagene). The sequences of the primer sets and the annealing temperatures are shown in Table S1.

### Generation of transgenic wheat lines

Transgenic wheat plants were produced by a particle bombardment method using immature embryos. The expression plasmid *35S:VRN3-D* was constructed and co-transformed with the plasmid pUBA (Toki *et al.*, 1992), which contains the *bar* gene as a selection marker under the control of the maize ubiquitin promoter, according to the method of Pellegrineschi *et al.* (2002). The integration of the transgene was confirmed by PCR of genomic DNA using primers designed from the 35S promoter sequence (35S-WFT-FW: 5′-CGCACAATCCCACTATCCTT-3′), and from the *WFT* cDNA sequence (35S-WFT-RV: 5′-GAAGAGCACGAGCACGAAG-3′). One transgenic plant, designated WF3, was selected from six independent transgenic T_0_ plants. Using 16 T_1_ plants derived from the WF3 T_0_ plant and two non-transgenic plants, RT-PCR experiments were carried out to examine the transcription levels of *FT*, *VRN1* and *VRN2*.

## Notes

Sequence data from this article can be found in the DDBJ/EMBL/GenBank data libraries under the accession number AB361064 for *WCO1-D*.

## SUPPORTING INFORMATION

**Figure S1.** PCR analysis for the determination of the chromosomal location of *WCO1*.

**Figure S2.** Alignment of the deduced amino acid sequences of *CO*-like genes.

**Figure S3.** PCR analysis for detecting *VRN2* alleles.

**Figure S4.** Sequence of promoter and coding regions of the *VRN3* gene in the *mvp* mutant.

**Figure S5.** Sequences of the RT-PCR products for the expression analysis of *FT* in transgenic plants.

**Table S1.** Sequences of PCR primer sets and annealing temperatures used in the expression analyses.
