# Supplementary figures and images for: A genetic network of flowering-time genes in wheat leaves, in which an APETALA1/FRUITFULL-like gene, VRN1, is upstream of FLOWERING LOCUS T
Source: Plant J. 2009 Feb 26;58(4):668–81. doi: 10.1111/j.1365-313X.2009.03806.x (PMC2721963; doi:10.1111/j.1365-313X.2009.03806.x)

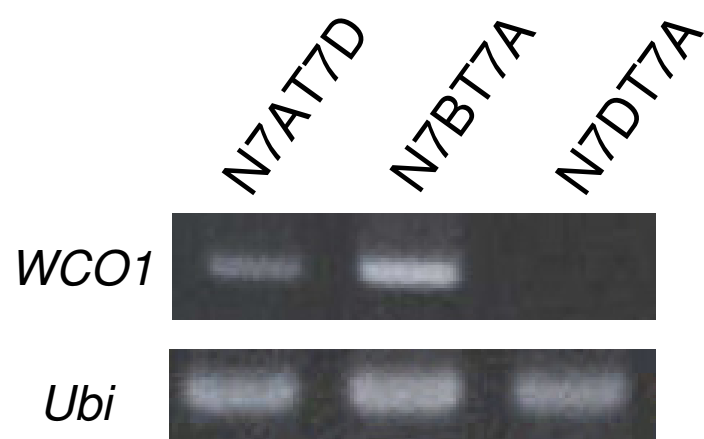

**Figure S1**

Supplement: Supplementary file 1 [file tpj0058-0668-SD1.pdf]

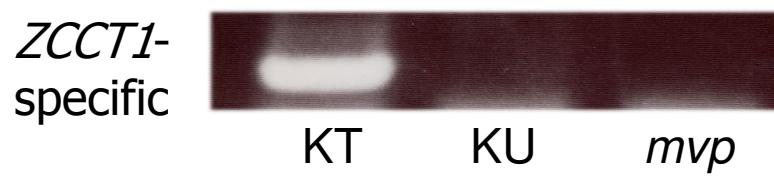

Figure S3

Supplement: Supplementary file 3 [file tpj0058-0668-SD3.pdf]
