# Supplementary material for: A genetic network of flowering-time genes in wheat leaves, in which an APETALA1/FRUITFULL-like gene, VRN1, is upstream of FLOWERING LOCUS T
Source: Plant J. 2009 Feb 26;58(4):668–81. doi: 10.1111/j.1365-313X.2009.03806.x (PMC2721963; doi:10.1111/j.1365-313X.2009.03806.x)
Supplement: Supplementary file 2 [file tpj0058-0668-SD2.pdf]

```
HvCO4     ------MEGEEKPVVGG---------AYWGVGARACDSCATEAAR-LFCRADAAFLCAGC
HvCO5     ------MKVEEQTVVGGGGGAGQGGAGFWGLAGRPCDTCAVDAAR-LYCRLDGAYLCAGC
HvCO6     ---------------------MMELRKYWGVGGRRCGGCEGAAPAAVHCRDCAGYLCTGC
HvCO7     ------------------------------------------------------------
HvCO9     ------------------------------------------------------------
HvCO3     --------------------------------------------------MIKAEPDLRG
HvCO8     -----------------------------------------------------------M
TaHd1     MFMNCNFNSNLLENEAGRISFP----------WARPCDGCHAAPSAVYCCADAAYLCASC
HvCO2     --MNCNFNSDLLEKEAGRTSFP----------WARPCDGCHAAPSAVYCCADAAYLCSSC
WCO1      --MNCVSNGTVYEEAVGREGS-----------WARLCDGCCTVPSVVYCRADSAYLCASC
HvCO1     --MNCVSNGTVYEEAVGREGR-----------WARLCDGCCTVPSVVYCRADSAYLCASC
Hd1       --MNYNFGGNVFDQEVGVGGEGGGGGEGSGCPWARPCDGCRAAPSVVYCRADAAYLCASC

HvCO4     DARAHGS---GSRHARVWLCEVCEHAPAAVTCKADAAVLCASCDADIHAANPLARRHERV
HvCO5     DARAHGA---GSRHARVWLCEVCEHAPAAVTCRADAAALCATCDADIHSANPLASRHLLL
HvCO6     DARPAHA---RAGHERVWVCEVCEVSPAAVTCKADAAVLCAACDADIHHANPLAERHVRV
HvCO7     ------------------------------------------------------------
HvCO9     ------------------------------------------------------------
HvCO3     QLRGSAG---VGGMQLQQRCDSCRSAPCAFYCRADSAALCAACDADVHSANTLASRHRRV
HvCO8     ESEGSTS---ANGGAACAVCG----GSAALYCPADAAALCVPCDAAVHSANPLASRHERV
TaHd1     DTQVHSANRVASRHERVRVCETCESAPAVLACHADAAALCTACDAQVHSANPIAQRHQRV
HvCO2     DTQVHSANRVASRHERVRVCETCESTPAVLACHADAAALCTACDAQVHSANPIAQRHQRV
WCO1      DAQIHAANRVASRHERVLLSEAYKHAPVVLECHADAAALCAAYEAQVHYANLLATMHQRV
HvCO1     DAQIHAANRVASRHERVLLSEAYKHAPVMLDCHADAAALCAAYEAQVHYANLLTVMHQRM
Hd1       DARVHAANRVASRHERVRVCEACERAPAALACRADAAALCVACDVQVHSANPLPAITIPA

HvCO4     PVAP--------------------------------------------------------
HvCO5     PTP---------------------------------------------------------
HvCO6     PIAPIGSPEAAAVAAEAMMLCGAGDGDARADPDEVHDQLHHHGHGGMLNLNVEAGKEGGK
HvCO7     ------------------------------------------------------------
HvCO9     ------------------------------------------------------------
HvCO3     PMGAVAPASPAGGAFVVRPGGVNSSWPIREGRRSYYDD-----------------REGEE
HvCO8     PLAAAVAVAATSG-----------------------------------------------
TaHd1     PVLPLPAVAIPAASGFAEAEASVTAHGDKEEGEEVDSWRLRRN-----SDDNNCAN----
HvCO2     PVLPLPAVAIPAASGFAEAEASVTAHGDKEGGEEVDSWLLRRN-----SDDNNCAN----
WCO1      PVVSHPVAAIPAASLFAEAAATAPVLGSKEEDASWLLLSKDSD-----NHNHSGNHSSSS
HvCO1     PVVSHPVAAIPPVSLFAEAEATAPVLGRKEEDTSWLLLSKDSD-----NHNRSGNNSSTS
Hd1       TSVLAEAVVATATVLGDKDEEVDSWLLLSKDSDNNNNNNNNND-----NDNNDNNNSNSS

HvCO4     ---FFGAAADAHKPFPSSGAQAGAAASAED--------DGSNDAEAASWLLPEPDHKDGA
HvCO5     ---FFGALADPPQPVPSPSSAAATQEDAED--------DGSNEAEAASWLLPEPGDSP--
HvCO6     MDYLFSDLVDPYLAVDFTRFAHADSVVPNG--------VATAAVPAVVDLDFACGIGAKP
HvCO7     -------MMDPFFGSELPRFPHADSVVPSG--------GAVELDFGGVAAAAAVVS----
HvCO9     ------------------------------------------------------------
HvCO3     EEATSWLLLDPLRGSEADAPAFGDALVADFL-------DLGRAGEKEASSKDYHGHGMES
HvCO8     -------VYDGLFAADDDGAASWPTPVQGSP-------NSGSSGFS-------NGSGVEV
TaHd1     -------KIDRYYNLVGYNMYYNNITCDPR---------PEEQYRMQEQ-RVQNRYIEKQ
HvCO2     -------KIDRYFNLVGYNMYYDNITCDPR---------PQEQYRMQEQQHVQNRYREKE
WCO1      SSSRYFGEVDQYFDLVGYNSYYDSHMNNQEQYVMQEQQHLQQMQKEYAEQQMQKEYVEKE
HvCO1     SSSQYFGEVDQYFDLVGYNSYYDSHMSNQEQYVMQEQQHLQQMQKEYAEQQMQKEYVENE
Hd1       NNGMYFGEVDEYFDLVGYNSYYDNRIENNQ---DRQYGMHEQQEQQQQQQEMQKEFAEKE

HvCO4     NGATADVFFADSDHYLDLDFARSMDDIKAISVQLNGQPEIDLNGGN----KGFYSDHSMN
HvCO5     -EDSAATFFADSDAYLDLDFVRSMDGIKAIGVPVAPS-ELDLAGG-----TLFYPEHSMN
HvCO6     PPSYSSSYTANGSGAHSGSSSEVGVVPEAIHG-GAGSFELDFTRPKPQAYMPAYTPAPPS
HvCO7     NPSYSSYTAASIG--GSGSSSEVGLVPDAICGRGGGIIELDFAQS--KAAYLPYTPTPSH
HvCO9     ------------------------------------------------------------
HvCO3     NEGSHDHELVVPGEPVAQLHERQGFTAEMAYDAQNSNHGYGFGATFERSLSMSSSPDNSS
HvCO8     S---------------------------------------LFDLLSDVDLVATGAGGSVS
TaHd1     GCECVVPPQVVMASEQQESDYG-TRGAGQAASVTAITSTYTASISNDISFSSMEVGIIPD
HvCO2     GCECVVPPQVVMASEQQGSNYG-TIGAGQAASVTAMASTYTASISNDISFSSMEVGIVPD
WCO1      GSECIVPSQSAIVSRPHQSGYAPLVRAEQAASVTAGVSAYTDSVNNSISFS-MEAGIVPD
HvCO1     GSECIVPSQSTIVRRPHQSGYAPLVGAEQAASATAGASAYTDSVNNSISFS-MEAGIVPD
Hd1       GSECVVPSQITMLSEQQHSGYG-VVGADQAASMTAGVSAYTDSISNSISFSSMEAGIVPD

HvCO4     HSLSSSEAAVVPDAAAAP-----------VVSRGREREARLMRYREKRKSRRFEKTIRYA
HvCO5     HSMSTSEVAVVPDALSAGGAPAPAPSVAVVASKGKEREARLMRYREKRKNRRFQKTIRYA
HvCO6     HGVGMQQASAVDMGYLTVPERP-------VAVTGEGRVARLMRYREKRKNRRFEKTIRYA
HvCO7     STVSSVDVGPVPERSESAVAAA-------TPAMGEGREARLMRYREKRKNRRFEKTIRYA
```

```
HvCO9           ------------------------------------REAKLMRYKEKRKRRRYEKQIRYA
HvCO3           TVQDVSSSYMRRSESSVDLFSTAAHTSPQFMGMAMDREARVHRYREKRKMRRFEKTIRYA
HvCO8           SDGGVAPLWLQPGLASAAWNSSWWRSEAVVVPSRPDGAARVMRYREKRKNRKFHKTIRYA
TaHd1           NTRPDISNSNILTGSEAMELSG-HSLQMPVHFSSMDREARVLRYKEKKQTRKFQKTIRYA
HvCO2           NTRPNISNRNILTSSEAIELSG-HSLQMPVHFSSMDREARVLRYKEKKQARKFQKTIRYA
WCO1            NTVQSS----ILRPAGAIGHFSSPSLQTPLHFSSKEREARVLRYKEKKKSRKFEKTTRYA
HvCO1           NTVQSS----ILRPAGAIGLFSSPSLQTPLHFSSKEREARVLRYKEKKKSRKFEKTTRYA
Hd1             STVIDMPNSRILTPAGAINLFSGPSLQMSLHFSSMDREARVLRYREKKKARKFEKTIRYE
                                                      *:: **:**:: *::.*  **

HvCO4           SRKAYAETRPRVKGRFAKRTGTADADALEEHEEMYSSAAAAVAALMAPGPDHDYGVDGVV
HvCO5           SRKAYAETRPRIKGRFAKRT--AEDDALEQDGPFSPASSAHLASDGDYG---------VV
HvCO6           SRKAYAESRPRVKGRFAKRADQDADGDGDDLDAEAHAVPSSTSYLLDFG-------YGVV
HvCO7           SRKAYAESRPRVKGRFAKRADDAD----ADAVAAGTITAPRPCVLDFSG-------YGVV
HvCO9           SRKAYAEMRPRVKGRFAKVPDGGEGAAPSPPQQPTQAAGYEPSRLDLGWFRS--------
HvCO3           SRKAYAETRPRIKGRFAKRADADLEVDQYFSAAALS-----------------DSSCGVV
HvCO8           SRKAYAEARPRLKGRFVKRPAAAATDDGNTSAAEAAKFWLSFSDNSVGFH---VASHGVV
TaHd1           TRKAYAEARPRIKGRFAKRSDIEHEEDHMLSPPALPDTSS----------------YNTV
HvCO2           TRKAYAEARPRIKGRFAKRSDIEHEENHMLSPPALPDTSS----------------YNTV
WCO1            TRKAYAEARPRIKGRFAKRSDADMEVDQTFSTAALSDSS-----------------YSTV
HvCO1           TRKAYAEARPRIKGRFAKRSDAEMEVDQTFSTAALSDSS-----------------YSTV
Hd1             TRKAYAEARPRIKGRFAKRSDVQIEVDQMFSTAALSDGS-----------------YGTV
                :****** ***:****.* .

HvCO4           PTLV
HvCO5           PSF-
HvCO6           PSF-
HvCO7           PTF-
HvCO9           ----
HvCO3           PTF-
HvCO8           PSF-
TaHd1           PWF-
HvCO2           PWF-
WCO1            PWF-
HvCO1           PWF-
Hd1             PWF-
```

Figure S2
