# Supplementary material for: A genetic network of flowering-time genes in wheat leaves, in which an APETALA1/FRUITFULL-like gene, VRN1, is upstream of FLOWERING LOCUS T
Source: Plant J. 2009 Feb 26;58(4):668–81. doi: 10.1111/j.1365-313X.2009.03806.x (PMC2721963; doi:10.1111/j.1365-313X.2009.03806.x)
Supplement: Supplementary file 4 [file tpj0058-0668-SD4.pdf]

<Promoter region>

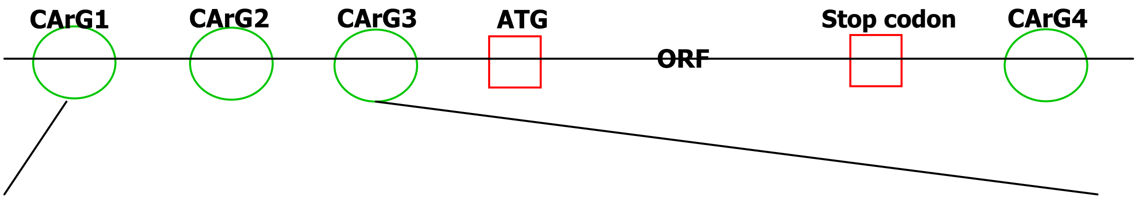


```
KU104-2    GTAAGCTAACGTTTCGGGAAAGTCTGTCTTCTTCATCCTTTCCGACCACGCACACACCCAGCAGCAAGAATGAACCCTGTCCTCCAATGATCTCCCCCGTACGACCCATCCGAGGCTGTG
mvp        GTAAGCTAACGTTTCGGGAAAGTCTGTCTTCTTCATCCTTTCCGACCACGCACACACCCAGCAGCAAGAATGAACCCTGTCCTCCAATGATCTCCCCCGTACGACCCATCCGAGGCTGTG
           ************************************************************************************************************************

KU104-2    TGATCTTGCTCTCCCTCCCCGTCGTCACCATGCACGTGAAACGGGCGGGCGGGCTTTCGACACCTTCCCAGCTACGGCCGGCGGCAGCTGATGAAGCTTACATCAAATCGAGCCAAGGAA
mvp        TGATCTTGCTCTCCCTCCCCGTCGTCACCATGCACGTGAAACGGGCGGGCGGGCTTTCGACACCTTCCCAGCTACGGCCGGCGGCAGCTGATGAAGCTTACATCAAATCGAGCCAAGGAA
           ************************************************************************************************************************

KU104-2    GCATGCACCCCAGTCACCGTCTCGCGCTAGCTAATTGGCAGACATTCCCTGTGCCCGCTTGCCGGCCGGCCGCGGCGTGACCGCCGGTCGGCCCAGAGCCCCGGTCGCAACGCAAACCTA
mvp        GCATGCACCCCAGTCACCGTCTCGCGCTAGCTAATTGGCAGACATTCCCTGTGCCCGCTTGCCGGCCGGCCGCGGCGTGACCGCCGGTCGGCCCAGAGCCCCGGTCGCAACGCAAACCTA
           ************************************************************************************************************************

                                                       CArG2
KU104-2    CACGCCAGCAGGAGCAGGCAGCCAGGCACGGCCTAGAAGCCACCATTAATTTGCGTGGTGATCATGATCAGGAGCTTATTACGGCAGACAGATGCATCCATCGGTCTCGCTTCTGCCTGT
mvp        CACGCCAGCAGGAGCAGGCAGCCAGGCACGGCCTAGAAGCCACCATTAATTTGCGTGGTGATCATGATCAGGAGCTTATTACGGCAGACAGATGCATCCATCGGTCTCGCTTCTGCCTGT
           ************************************************************************************************************************

KU104-2    GGGGGTCAAAAGCGCTGCCGGTTGTACCACGTCCACAGAACCAATTCAATAGAGAGAGGCGACGAGATTCCGTGGCCACGCCAGCTCGGCAGCGCCAAGGAGTACTAGAGCGGAGAGCAG
mvp        GGGGGTCAAAAGCGCTGCCGGTTGTACCACGTCCACAGAACCAATTCAATAGAGAGAGGCGACGAGATTCCGTGGCCACGCCAGCTCGGCAGCGCCAAGGAGTACTAGAGCGGAGAGCAG
           ************************************************************************************************************************

KU104-2    CGGCTGAACTGGTCTGGACATGGACATGGACATGGACATGGAACATGCCAGGCTGAGCTTT
mvp        CGGCTGAACTGGTCTGGACATGGACATGGACATGGACATGGAACATGCCAGGCTGAGCTTT
           *************************************************************
```

<Coding region>

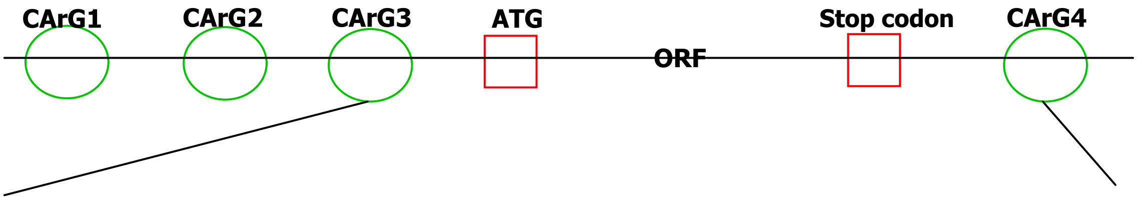


```
KU104-2   TCCTCAATTCACAGCTTACGCTTACTCCTGCTCCAGATAACCTCTGCTGCTTGCTCCCTCTCGTACCCTAGCAGCTAGCTAGCCGGTCGATCTACACTAGGAAGAAGGAAGGGGAAATGG

mvp       TCCTCAATTCACAGCTTACGCTTACTCCTGCTCCAGATAACCTCTGCTGCTTGCTCCCTCTCGTACCCTAGCAGCTAGCTAGCCGGTCGATCTACACTAGGAAGAAGGAAGGGGAAATGG

          ************************************************************************************************************************


KU104-2   CCGGGAGGGACAGGGACCCGCTGGTGGTTGGCAGGGTTGTGGGGGACGTGCTGGACCCCTTTGTCCGGACCACCAACCTCAGGGTGACCTTCGGGAACAGGACCGTGTCCAACGGCTGCG

mvp       CCGGGAGGGACAGGGACCCGCTGGTGGTTGGCAGGGTTGTGGGGGACGTGCTGGACCCCTTTGTCCGGACCACCAACCTCAGGGTGACCTTCGGGAACAGGACCGTGTCCAACGGCTGCG

          ************************************************************************************************************************


KU104-2   AGCTCAAGCCGTCCATGGTCGCCCAGCAGCCCAGGGTTGAGGTGGGCGGCAATGAGATGAGGACCTTCTACACACTCGTACGTACACAGTCACTATCTAATGCCTATATGTTAAGCTCTG

mvp       AGCTCAAGCCGTCCATGGTCGCCCAGCAGCCCAGGGTTGAGGTGGGCGGCAATGAGATGAGGACCTTCTACACACTCGTACGTACACAGTCACTATCTAATGCCTATATGTTAAGCTCTG

          ************************************************************************************************************************


KU104-2   AAAGTGCTCGCCACACGCACATGATCGATCGGGCTCTATATATAGTACGTGCGGGAAGATGATTATCGATGCTTCTGTTCACAGCATGTTTGTCTTGGCAGGCACATGACTAATGCTCCA

mvp       AAAGTGCTCGCCACACGCACATGATCGATCGGGCTCTATATATAGTACGTGCGGGAAGATGATTATCGATGCTTCTGTTCACAGCATGTTTGTCTTGGCAGGCACATGACTAATGCTCCA

          ************************************************************************************************************************


KU104-2   TCTTGCATATGGCTCTGTGCTAGCTCTCTGGTGTTCATCATGATTTTCTATGCTTCTTTTCTATTCGGGGAACACTGATTTTCGATGCTTCTGTTGACATGTTTTATGTTTGTTCTGGCA

mvp       TCTTGCATATGGCTCTGTGCTAGCTCTCTGGTGTTCATCATGATTTTCTATGCTTCTTTTCTATTCGGGGAACACTGATTTTCGATGCTTCTGTTGACATGTTTTATGTTTGTTCTGGCA

          ************************************************************************************************************************


KU104-2   AGCACACGACTAATTAAAGCTCGATCTTAAATATATGCTTATGCACGTAGTACTCTCTACATCTCTAGTATTGATCATGATGTGCACGCGTGTACTGCCCGCAGGGGATGGTAGACCCAG

mvp       AGCACACGACTAATTAAAGCTCGATCTTAAATATATGCTTATGCACGTAGTACTCTCTACATCTCTAGTATTGATCATGATGTGCACGCGTGTACTGCCCGCAGGGGATGGTAGACCCAG

          ************************************************************************************************************************


KU104-2   ATGCTCCAAGTCCAAGCGATCCCAACCTTAGGGAGTATCTCCACTGGTAAGTAAATTTGTAGCTCAGTTGAATAATTTCTCTTTCCCTAGATATACACACTAGCTCATGTGTGTGTGTGT

mvp       ATGCTCCAAGTCCAAGCGATCCCAACCTTAGGGAGTATCTCCACTGGTAAGTAAATTTGTAGCTCAGTTGAATAATTTCTCTTTCCCTAGATATACACACTAGCTCATGTGTGTGTGTGT

          ************************************************************************************************************************
```

```
KU104-2    GTGCACGCGCGTGTGCATCTACATGTGTGTGCAGGCTTGTGACAGATATCCCCGGTACACTGGTGCCTCGTTCGGGCAGGAAGTGATGTGCTATGAGAGCCCTCGTCCGACCATGGGGAA

mvp        GTGCACGCGCGTGTGCATCTACATGTGTGTGCAGGCTTGTGACAGATATCCCCGGTACAACTGGTGCCTCGTTCGGGCAGGAAGTGATGTGCTATGAGAGCCCTCGTCCGACCATGGGGA

           ************************************************************************************************************************


KU104-2    TCCACCGCTTCGTGCTCGTGCTCTTCCAGCAGCTCGGCCGGCAGACCGTGTACGCCCCCGGGTGGCGCCAGAACTTCAACACCAGGGACTTCGCCGAGCTCTACAACCTCGGCCCGCCCG

mvp        TCCACCGCTTCGTGCTCGTGCTCTTCCAGCAGCTCGGCCGGCAGACCGTGTACGCCCCCGGGTGGCGCCAGAACTTCAACACCAGGGACTTCGCCGAGCTCTACAACCTCGGCCCGCCCG

           ************************************************************************************************************************


KU104-2    GGTGGCGCCAGAACTTCAACACCAGGGACTTCGCCGAGCTCTACAACCTCGGCCCGCCCGTCGCCGCCGTCTACTTCAACTGCCAGCGTGAGGCCGGCTCCGGTGGCAGGAGGATGTACG

mvp        GGTGGCGCCAGAACTTCAACACCAGGGACTTCGCCGAGCTCTACAACCTCGGCCCGCCCGTCGCCGCCGTCTACTTCAACTGCCAGCGTGAGGCCGGCTCCGGTGGCAGGAGGATGTACG

           ************************************************************************************************************************


KU104-2    AATTGACCTACCCATGGCCCACGTACGCCACCCGCAAAGTCAGCAACTTATCCAACGTGGCTAGTTTACTAGT

mvp        AATTGACCTACCCATGGCCCACGTACGCCACCCGCAAAGTCAGCAACTTATCCAACGTGGCTAGTTTACTAGT

           *************************************************************************
```

Figure S4
