# Supplementary material for: A genetic network of flowering-time genes in wheat leaves, in which an APETALA1/FRUITFULL-like gene, VRN1, is upstream of FLOWERING LOCUS T
Source: Plant J. 2009 Feb 26;58(4):668–81. doi: 10.1111/j.1365-313X.2009.03806.x (PMC2721963; doi:10.1111/j.1365-313X.2009.03806.x)
Supplement: Supplementary file 5 [file tpj0058-0668-SD5.pdf]

```
VRN3-A   1:AAG-AAGGAAGGGGAAATGGCCGGGAGGGACAGGGACCCGCTGGTGGTTGGCAGGGTTGT 59
VRN3-B   1:TAGGAAGGAAGGGCTAATGGCCGGTAGGGATAGGGACCCGCTGGTGGTTGGCAGGGTTGT 60
VRN3-D   1:TAAGAAGGAAGGGGGAATGGCCGGGAGGGACAGAGACCCGCTGGTGGTTGGCAGGGTTGT 60
WFT4     1:TAAGAAGGAAGGGGGAATGGCCGGGAGGGACAGAGACCCGCTGGTGGTTGGCAGGGTTGT 60
WFT5     1:TAAGAAGGAAGGGGGAATGGCCGGGAGGGACAGAGACCCGCTGGTGGTTGGCAGGGTTGT 60

VRN3-A  60:GGGAGACGTGCTGGACCCCTTTGTCCGGACCACCAACCTCAGGGTGACCTTCGGGAACAG 119
VRN3-B  61:GGGGGACGTGCTGGACCCCTTCGTCCGGACCACCAACCTCAGGGTGACCTTCGGGAACAG 120
VRN3-D  61:GGGGGACGTGCTGGACCCCTTCATCCGGACCACCAACCTCAGGGTGACCTTCGGGAACAG 120
WFT4    61:GGGGGACGTGCTGGACCCCTTCATCCGGACCACCAACCTCAGGGTGACCTTCGGGAACAG 120
WFT5    61:GGGGGACGTGCTGGACCCCTTCATCCGGACCACCAACCTCAGGGTGACCTTCGGGAACAG 120

VRN3-A120:GACCGTGTCCAACGGCTGCGAGCTCAAGCCGTCCATGGTCGCCCAGCAGCCCAGGGTTGA 179
VRN3-B121:GACCGTGTCCAACGGCTGCGAGCTCAAGCCGTCCATGGTCGCCCAGCAGCCCAGGGTTGA 180
VRN3-D121:GACCGTGTCCAACGGCTGCGAGCTCAAGCCGTCCATGGTCGCCCAGCAGCCCAGGGTTGA 180
WFT4   121:GACCGTGTCCAACGGCTGCGAGCTCAAGCCGTCCATGGTCGCCCAGCAGCCCAGGGTTGA 180
WFT5   121:GACCGTGTCCAACGGCTGCGAGCTCAAGCCGTCCATGGTCGCCCAGCAGCCCAGGGTTGA 180

VRN3-A180:GGTGGGCGGCAATGAGATGAGGACCTTCTACACACTCGTGATGGTAGACCCAGATGCTCC 239
VRN3-B181:GGTGGGCGGCAATGAGATGAGGACCTTCTACACACTCGTGATGGTAGACCCAGATGCTCC 240
VRN3-D181:GGTGGGCGGCAATGAGATGAGGACCTTCTACACACTCGTGATGGTAGACCCAGATGCTCC 240
WFT4   181:GGTGGGCGGCAATGAGATGAGGACCTTCTACACACTCGTGATGGTAGACCCAGATGCTCC 240
WFT5   181:GGTGGGCGGCAATGAGATGAGGACCTTCTACACACTCGTGATGGTAGACCCAGATGCTCC 240

VRN3-A240:AAGTCCAAGCGATCCCAACCTTAGGGAGTATCTCCACTGGCTTGTGACAGATATCCCCGG 299
VRN3-B241:AAGTCCAAGCGATCCCAACCTTAGGGAGTATCTCCACTGGCTTGTGACAGATATCCCCGG 300
VRN3-D241:AAGTCCAAGCGATCCCAACCTTAGGGAGTATCTCCACTGGCTTGTGACAGATATCCCCGG 300
WFT4   241:AAGTCCAAGCGATCCCAACCTTAGGGAGTATCTCCACTGGCTTGTGACAGATATCCCCGG 300
WFT5   241:AAGTCCAAGCGATCCCAACCTTAGGGAGTATCTCCACTGGCTTGTGACAGATATCCCCGG 300

VRN3-A300:TACAACTGGTGCCTCGTTCGGGCAGGAAGTGATGTGCTATGAGAGCCCTC           349
VRN3-B301:TACAACTGGTGCGTCGTTCGGGCAGGAGGTGATGTGCTACGAGAGCCCTC           350
VRN3-D301:TACAACTGGTGCATCCTTCGGGCAGGAGGTGATGTGCTACGAGAGCCCTC           350
WFT4   301:TACAACTGGTGCATCCTTCGGGCAGGAGGTGATGTGCTACGAGAGCCCTC           350
WFT5   301:TACAACTGGTGCATCCTTCGGGCAGGAGGTGATGTGCTACGAGAGCCCTC           350
```

primer

Figure S5
