# Supplementary material for: A genetic network of flowering-time genes in wheat leaves, in which an APETALA1/FRUITFULL-like gene, VRN1, is upstream of FLOWERING LOCUS T
Source: Plant J. 2009 Feb 26;58(4):668–81. doi: 10.1111/j.1365-313X.2009.03806.x (PMC2721963; doi:10.1111/j.1365-313X.2009.03806.x)
Supplement: Supplementary file 6 [file tpj0058-0668-SD6.pdf]

Table S1. Sequences of PCR primer sets and annealing temperatures used in the expression analyses

Primer name	Sequence (5'-3')	Annealing temp. (℃)
(RT-PCR analysis)		
VRN1-BAC 81655L	TCGTGGAGAAGCAGAAGGC	60
VRN1-BAC 82017R	GTTGATGTGGCTCACCATCC	
WCO1-L	GCACCACTTGTAGGGGCAGA	65
WCO1-R	TTGATCCTTGGCCGTGCTT	
WFT-all-3L	GGAGGTGATGTGCTACGAGAG	55
WFT-all-3R	CACACAAGAGTCAAGACCAACC	
Ubi-1L	GCATGCAGATATTTGTGAA	55
Ubi-1R	GGAGCTTACTGGCCAC	
WFT-FW	TAAGAAGGAAGGGGGAATGG	58
WFT-RV	GAGGGCTCTCGTAGCACATC	
WAP1-556L	ATCAGACTCAGCCTCAAACA	55
WAP1-982R	TAGAGACGGGTATCATGGAA	
VRN2-FW2	ATCGTCATCACCATCATCAG	58
VRN2-RV2	GTCATGATTGCTTCATTGCT	
ACT-FW	GGTACTCCCTCACAACAACC	58
ACT-RV	CCAGGAACTTCCATACCAAC	
(real-time PCR analysis)		
TaGI-3L	GAAGGTCAGAAGATGTGGAGAGTCAAC	63
TaGI-3R	GGCAGCGGATGGTAGGTGATAG	
TaHd1-2L	CCAGTACCTACACAGCTTCCA	63
TaHd1-2R	GCCTGCTTCTTCTCCTTGT	
WFT-F4	CAGGCCGGTCGATCTATACTA	58
WFT-R4	TCCTGTTCCCGAAGGTCA	
WAP1-545L	GGAGAGGTCACTGCAGGAGGA	65
WAP1-698R	GCCGCTGGATGAATGCTG	
ZCCT1-1Lt	GGCCTGCCCATTATCCC	65
ZCCT1-1Rt	GCGAAGCTGGAGATGATGG	
actin361-L	TATGCCAGCGGTCGAACAAC	58
actin361-R	GGAACAGCACCTCAGGGCAC	
